# Titanium versus polyetheretherketone versus structural allograft in anterior cervical discectomy and fusion: A systematic review

**DOI:** 10.1016/j.bas.2022.100923

**Published:** 2022-08-22

**Authors:** Jacob L. Goldberg, Ross M. Meaden, Ibrahim Hussain, Pravesh S. Gadjradj, Danyal Quraishi, Fabian Sommer, Joseph A. Carnevale, Branden Medary, Drew Wright, K. Daniel Riew, Roger Hartl

**Affiliations:** a Department of Neurological Surgery, Weill Cornell Medical Center, New York Presbyterian. New York, New York, USA; b Department of Library Information Technologies and Services, Weill Cornell Medicine, New York, NY, USA

**Keywords:** Anterior cervical discectomy and fusion, Pseudarthrosis, Titanium, PEEK, Structural allograft, Systematic review, Anterior cervical discectomy and fusion (ACDF), Polyetheretherketone (PEEK), randomized control trial (RCT), prospective cohort (PC), retrospective cohort (RC), patient reported outcome (PRO), neck disability index (NDI), visual analog scale (VAS)

## Abstract

**Introduction:**

Anterior cervical discectomy and fusion (ACDF) is a common procedure to address cervical spine pathology. The most common grafts used are titanium, polyetheretherketone (PEEK), or structural allograft. Comparison of fusion rate is difficult due to non-standardized methods of assessment. We stratified studies by method of fusion assessment and performed a systematic review of fusion rates for titanium, PEEK, and allograft.

**Research question:**

Which of the common implants used in ACDF has the highest reported rate of fusion?

**Materials and methods:**

An experienced librarian performed a five-database systematic search for published articles between 01/01/1990 and 08/07/2021. Studies performed in adults with at least 1 year of radiographic follow up were included. The primary outcome was the rate of fusion. Fusion criteria were stratified into 6 classes based upon best practices.

**Results:**

34 studies met inclusion criteria. 10 studies involving 924 patients with 1094 cervical levels, used tier 1 fusion criteria and 6 studies (309 patients and 367 levels) used tier 2 fusion criteria. Forty seven percent of the studies used class 3–6 fusion criteria and were not included in the analysis. Fusion rates did differ between titanium (avg. 87.3%, range 84%–100%), PEEK (avg. 92.8%, range 62%–100%), and structural allograft (avg. 94.67%, range 82%–100%).

**Discussion and conclusion:**

After stratifying studies by fusion criteria, significant heterogeneity in study design and fusion assessment prohibited the performance of a meta-analysis. Fusion rate did not differ by graft type. Important surgical goals aside from fusion rate, such as degree of deformity correction, could not be assessed. Future studies with standardized high-quality methods of assessing fusion, are required.

## Highlights

• Anterior cervical discectomy and fusion (ACDF) is a common procedure to address cervical spine pathology.
• The optimal graft type of promote fusion is unknown. The methods for assessing fusion are not standardized.
• This is a systematic review comparing fusion rates for ACDF grafts after first stratified by method of fusion assessment.
• Significant heterogeneity in study design and fusion assessment prohibited the performance of a meta-analysis; Fusion rate did not differ by graft type.
• Future studies with standardized high-quality methods of assessing fusion, are required.

## Funding

None.

## Registration

Prospero ID: CRD42021287457.

## 1 Introduction

Anterior cervical discectomy and fusion (ACDF) is a common procedure to treat radiculopathy or myelopathy caused by compression of the cervical spinal cord or nerve roots (Chin-See-Chong et al., 2017). Historically, iliac crest derived autograft was used as a substrate in the interbody space to facilitate bony fusion (Cloward, 1958). While once considered the gold standard, autograft use has declined due to procedure-related morbidity (Arrington et al., 1996). Additionally, synthetic interbody grafts can be used to correct malalignment, achieve higher rates of fusion, and provide indirect nerve root decompression (Hosoi et al., 2017).

Three commonly used graft materials include titanium, polyetheretherketone (PEEK), and cadaveric structural allograft. Each graft material has theoretical advantages and disadvantages inherent to its physical properties including modulus of elasticity, promotion or inhibition of osteogenesis, and visualization on post-operative imaging (Panayotov et al., 2016; Chong et al., 2015). Consensus regarding the optimal material for interbody graft has not been reached (Yoon et al., 2017). In part, this is due to non-standardized and subjective radiographic methods of fusion assessment (Oshina et al., 2018).

In this study, we sought to compare the reported fusion rates after ACDF based on graft material (titanium, PEEK, or structural allograft).

## 2 Methods

Institutional review board approval was not required as this study is a literature review. This study was designed and conducted in accordance with the Preferred Reporting Items for Systematic Reviews and Meta-Analyses (PRISMA) guidelines (Moher et al., 2009). This study was registered in the international prospective register of systematic reviews (Prospero CRD42021287457).

### 2.1 Search strategy

A multi-database (PubMed, MEDLINE, Embase, Cochrane, and Scopus) literature search was designed and conducted by a medical librarian (DW). Backward searches (snowballing) of reference lists of identified articles and earlier systematic reviews and forward searches (citation tracking) were conducted. As there has been significant evolution of implants, we only included studies from 1990 or later using non-experimental titanium cage design. Therefore, the search was performed from 1/1/1990 to 8/7/2021. Search concepts included anterior cervical discectomy and fusion, allograft, titanium, and polyetheretherketone. Comprehensive search strategies are detailed in Appendix 1. Two reviewers independently screened the identified studies based on the inclusion and exclusion criteria. A third reviewer served as the tie breaker when disagreement existed between the two primary reviewers. To facilitate the screening, Covidence Systematic Review Software (Veritas Health, Australia) was used for the screening of potentially relevant studies.

### 2.2 Inclusion and exclusion criteria

Inclusion criteria were studies reporting ACDF using either structural allograft, PEEK, or titanium interbody devices in adults for degenerative cervical indications. The studies had to specify their methods of assessing fusion with at least 1 year follow up. Study design included randomized controlled trials (RCTs), prospective (PC) or retrospective cohort (RC) studies. For studies in which only one treatment arm met inclusion criteria, data was extracted from that study arm alone. Exclusion criteria included non-original or peer reviewed studies, incomplete fusion data, and corpectomy. Studies without anterior plating or stand-alone cages without integrated instrumentation were excluded. Studies that used additional fusion augmentation measures such as bone morphogenic protein, stem cells, platelets, or supplemental posterior instrumentation to remove the potential confounding effects.

### 2.3 Fusion rate assessment

The most common methods of radiographic fusion assessment were stratified into a predetermined 6-tiered hierarchy based on a previous systematic review of cervical spine fusion criteria (Table 1) (Oshina et al., 2018). All studies meeting the inclusion criteria were categorized based on this hierarchy by two independent reviewers with a third reviewer serving as the tie breaker. When study criteria did not perfectly fit into the 6 described tiers, reviewers categorized them according to the tier most alike the methods described. Of the above tiers, studies in tiers 1 or 2 were deemed suitable for inclusion. All included studies, exact fusion criteria, and assigned fusion criteria tier are listed in Table 2.

**Table 1:** Classification of radiographic fusion criteria.

Tier 1	CT scan with confirmed extra-cage bridging bone or fused facets
Tier 2	Flexion-extension radiographs magnified >150% with <1 ​mm interspinous process motion at the fused levels and >4 ​mm motion at an adjacent non-fused level
Tier 3	CT with no described criteria
Tier 4	Flexion-extension radiographs with >1 ​mm interspinous process motion at the fused segment
Tier 5	Flexion-extension radiographs with no described criteria
Tier 6	No information provided on method of fusion assessment

**Table 2:** Study specific fusion criteria and assigned fusion criteria tier.

Author, Year Published	Fusion Criteria (Tier)	Definition of Fusion used in study
Douglas Moreland et al., 2004	1	Flexion/extension films were obtained. If there was a question of failed fusion, a CT scan was obtained. Fusion was defined by the following radiologic observations: the cage appears to be in a stable position, flexion/extension films show no motion or separation of the spinous process and no evidence of a radiolucent halo around the cage.
Sen Yang et al., 2019	1	We define fusion as… 1. The presence of bony trabeculation across the fusion level (bony bridging) and a lack of bony lucency at the juncture of the cage and vertebral body. 2. The absence of such bridges or the presence of an anterior–posterior discontinuation was classified as non-fusion.
Praveen Mummaneni et al., 2007	1	Radiographic evidence of fusion was based on the following criteria: 1. Bone spanning the two VBs in the treated segment. 2. Less than 4° of motion on dynamic radiographs; and 3. Radiolucencies covering no more than 50% of the implant surface.
Wai-MunYue et al., 2005	1	1. Absence of radiolucent lines at the interface between the allograft and the adjacent vertebral endplates, presence of bridging trabeculae across the entire interface, absence of motion of the adjacent vertebral bodies in flexion-extension radiographs, and presence of bridging bone in the intervertebral space beyond the limits of the allograft were assessed to determine fusion. 2. Graft incorporation was detected by comparing the radiodensity of the graft vis-a-vis adjacent vertebral bodies. 3. Lateral, flexion, and extension radiographs of their cervical spines were obtained to determine the fusion status and the presence of any late implant or graft complications.
Mitchell Campbell et al., 2009	1	Radiographs included upright anteroposterior, lateral, and flexion-extension radiographs. Fusion success was defined as the presence of bridging trabecular bone as evidenced by continuous bony connection of the vertebral bodies above and below in at least one of the following areas: lateral, anterior, posterior, and/or through the allograft ring implant; angulation of less than 4° on flexion-extension radiographs; and absence of radiolucency covering more than 50% of either the superior or inferior surface of the graft. The radiographs were reviewed by 2 independent radiologists who were blinded to which bracing group they were evaluating. A third independent adjudicate reviewer was used as needed.
Shang-Wen Feng et al., 2018	1	Plain radiographs of the cervical spine (anterior–posterior view, lateral view, flexion–extension view) were taken at 1, 3, 6, 12, and 24 months postoperatively. A computerized tomography (CT) scan was taken 12 months after the operation to evaluate the status of fusion. If fusion is not confirmed at 12 months, another CT scan will be arranged at 24 months postoperatively. Fusion status was assessed in the window at a setting of 420/40, 120 ​kV, 60–200 ​mA (Toshiba, Aquilion, Tokyo, Japan) to optimize the trabecular bone detail. The fusion was defined as follows: (1) rotation <4° and <1.25 ​mm translation with the absence of motion adjacent to interspinous processes (>3 ​mm) in the flexion-extension view and (2) the presence of continuous trabecular bone bridging was revealed by CT scan in at least one of the following locations: anterior, within, or posterior to the PEEK cage. A radiologist and a senior spine surgeon evaluated the fusion status independently without any preconceptions regarding patients' clinical outcomes. A fused status was recorded only when both reviewers agree.
Michael Hisey et al., 2015	1	Pseudoarthrosis defined as at least 2 degrees of angular motion on flexion/extension, or radiolucencies at >50% of the graft vertebral body interface, or absence of bridging bone across the graft vertebral body interfaces
Young-Seok Lee et al., 2014	1	Bridwell grading scale. The system is based on plain radiographic findings and state of fusion is divided into one of four grades. Grade I is defined as fusion with remodeling and trabeculae present; Grade II is an intact graft with incomplete remodeling and no lucency present; Grade III is an intact graft with potential lucency at the cranial or caudal end; Grade IV is absent fusion with collapse/resorption of the graft.
Jan-Helge Klingler et al., 2014	1	Fusion was determined in three-dimensional reconstructed CT scans and confirmed if continuous trabecular bone bridges through or around the implant were clearly present.
Ing How Moo et al., 2020	1	1) the interspinous distance (lack of movement at the operated levels with interspinous process motion having a < 1 ​mm difference in flexion and extension on an adequate scan, which was defined as the presence of interspinous process motion of at least 4 ​mm at the uninvolved adjacent segment), 2) the presence of a bridging bone across the fusion level observed on a computed tomography (CT) scan or a plain radiograph at the last follow-up, and 3) the absence of radiolucency at the graft–vertebral junction.
Toru Yamagata et al., 2012	1	Osseous bony fusion was evaluated based on a plain radiograph or sagittal CT images and classified as solid osseous fusion, partial fusion or no fusion. Solid osseous fusion was defined as a clear osseous bridge in the intervertebral space or inside the cage. Partial fusion was defined as incomplete osseous fusion without any instability.
Matthew Gornet et al., 2017	1	1) angulation ≤4°, 2) bridging bone as a continuous bony connection with the vertebral bodies above and below, and 3) no radiolucency covering more than 50% of either the superior or inferior surface of the graft.
Domagoj Coric et al., 2011	1	1) bridging trabecular bone; 2) angular motion less than 5°; 3) translational motion less than 3 ​mm; and 4) less than 50% radiolucency along the bone-implant interface.
You-Sub Kim et al., 2018	1	Fusion was assessed with both flexion–extension lateral radiographs and cervical computed tomography (CT) scans. We defined fusion when the following two conditions were satisfied: (i) ​< ​2 ​mm gap between the tips of the spinous process on flexion–extension lateral radiographs and (ii) partial or complete bony bridging on CT scans but not on lateral radiographs. We considered fusion type III or IV as a successful fusion.
Majid R. Farrokhi et al., 2017	1	Good - No motion on flexion/extension radiographs, no radiolucent zones between the cage and vertebrae, and trabecular bridging at both endplates. Average - No motion on flexion/extension radiographs, no radiolucent zones between the cage and vertebrae. Poor - Motion on flexion/extension radiographs, radiolucent zones between the cage and vertebrae, and no trabecular bridging at both endplates.
Yu Chen et al., 2013	2	1. Absence of motion between the spinous processes at dynamic lateral radiographs, 2. Absence of a radiolucent gap between the graft and endplates, 3. Presence of continuous bridging bony trabeculae at the graft-endplate interface.
Mario Cabraja et al., 2012	2	1. Movement of less than 2° was measured, and by the absence of motion between the spinous processes on lateral flexion-extension radiographs. 2. Movement of ≥2° on flexion/extension radiographs was regarded as a pseudarthrosis.
Kirsten Schmieder et al., 2006	2	The fusion mass was assessed on the lateral plain radiographs and two criteria were used: 1. The presence of bone bridges anterior and/or posterior to the cage. 2. The disappearance of the bone borders around the cages.
S.M. Rohe et al., 2009	2	The degree of fusion was assessed using the lateral plain radiographs and two criteria were used… 1. The presence of bone bridges anterior and/or posterior to the cage 2. The disappearance of the bone borders around the cages.
Shiuh-Lin Hwang et al., 2007	2	Successful fusion was defined according to the following criteria: 1. The absence of motion between spinous process on flexion-extension radiographs and the absence of any dark halo around a cage or iliac bone graft on both anteroposterior and lateral radiographies, 2. Or presence of bridging bone anterior or posterior to the cage or iliac bone graft
Wen-Jian Wu et al., 2012	2	Fusion was defined as a lack of motion between the vertebral bodies and cages on flexion/extension radiographs and the absence of any dark halo around a cage on both anteroposterior and lateral radiographs; or bone bridging the intervertebral space through or around the cage
Dino Samartsiz, 2005	2	Radiographic fusion was assessed on… lateral neutral, flexion, and extension views, and was established by the presence of a bony bridge incorporating the graft and adjacent endplates and when neither instrumentation motion nor radiolucencies were evident encompassing the screws.
Shiuh-Lin Hwang et al., 2005	2	Radiographic assessment of fusion was based on the presence or absence of motion between the spinous processes of the fused levels on flexion–extension views. Successful fusion was defined according to the following criteria: (1) absence of motion on flexion–extension radiographs and absence of any dark halo around a cage on both anteroposterior and lateral radiographs; or (2) presence of bridging bone anterior or posterior to the cage
Minghao Wang et al., 2020	3	1. Pseudarthrosis was counted if there was observation of motion on dynamic radiographs (change in vertebral body angulation or interspinous process distance), lucency through the fusion mass, the appearance of a halo around the screws, or implant failure that could be visualized on radiographs. 2. Thus, fusion was assessed by 2 independent spine surgeons, with a CT scan if available, and with dynamic radiographs assessing motion or stability of spinous process splaying. Only revision surgery for pseudarthrosis was recorded. Other causes unrelated to pseudarthrosis such as hematoma or infection were not included in the revision surgery rates.
Yu-Cheng Chou et al., 2008	3	All participants were followed-up via X-ray of the cervical spine and observation of clinical symptoms 6 and 12 months postoperatively. Standard, flexion-extension and bilateral oblique X-rays were obtained. Fusion was deemed to have occurred if trabecular bone appeared across the interfaces. Nonunion was deemed to have occurred if there was lucency between the implants and vertebral endplate surfaces
Ehab Shiban et al., 2016	3	Fusion was defined as the absence of motion between the spinous processes on flexion-extension radiographs. The measurement of interspinous distance on dynamic radiographs of≥2 ​mm was defined as non-fusion
Mark Arts et al., 2020	4	Fusion for this study was defined as rotation ≤4° and ≤1.25 ​mm translation on flexion-extension films at the index level. For comparison, we also calculated fusion status when range of motion (ROM) was <2° and <1°.
Cladius Thomé et al., 2004	4	The films were aligned so as to superimpose vertebral bodies for determining there was less than 2 degrees of segmental movement on lateral F/E views, the presence or absence of motion. Each operative segment was deemed fused if less than 50% of the anteroposterior distance of the interface between the endplates and implants was radiolucent, and if the interspinous distance did not change more than 2 ​mm. Two degrees and 2 ​mm of motion were used as the upper limits to compensate for experimental error and variation.
Cladius Thomé et al., 2006	4	Each surgically treated segment was deemed fused if there was obvious bridging bone, if there was less than 2° of segmental motion, and if the interspinous distance did not change by more than 2 ​mm. Two degrees and 2 ​mm of motion were used as the upper limits to compensate for measurement and radiographic projection error. If there was a lucent line at the implant's margin(s), the segment was considered to be unfused regardless of the aforementioned measurements. In patients in whom bisegmental surgery was performed, the mass was categorized as fused only if both levels met the aforementioned criteria.
Domagoj Coric, 2013	4	Fusion was defined using a composite measure consisting of greater than 50% trabecular bridging bone, 2° of motion or less, and no implant loosening. Range of motion was determined from dynamic lateral radiographs using the Philips iSite angle measurement tool. Bridging ossification in the cervical TDR group was defined as the presence of heterotopic bone with less than 2° of motion.
Der-Yang Cho et al., 2004	5	The criteria for bone fusion were set up as follows: bony specula across the fusion level on X-ray film and no change in position of fusion levels as seen by dynamic view (flexion or extension). If fusion diagnosis was questionable, patients underwent a thin-slide CT scan. If a radiolucent space existed between the graft and endplate after 1 year of follow-up, the fusion was incomplete (non-fusion, or pseudoarthrosis). If the graft height was deformed more than one-third of normal disc height, then kyphosis developed and the graft was defined as graft collapse.
Muhammad Junaid et al., 2018	6	Fusion Criteria not discussed/unclear
Der-Yang Cho et al., 2003	6	We followed the patients 6 months postoperatively with x-ray examinations to assess the fusion rate

### 2.4 Data collection and analysis

Two reviewers extracted data independently from the included studies. Extracted data included first author, publication year, study design, type(s) of interbody, number of patients, patient age, surgical indication, level(s), duration of follow up, fusion rate, reoperation rate, pseudarthrosis rate, subsidence rate. The patient reported outcomes (PROs) of neck disability index (NDI) and visual analog scale (VAS) were collected when pre- and post-operative values were available.

The primary outcome of this study is the reported interbody fusion rates. Secondary outcomes included cage subsidence, reoperation, and pseudoarthrosis.

## 3 Results

### 3.1 Search results

**Fig. 1:**
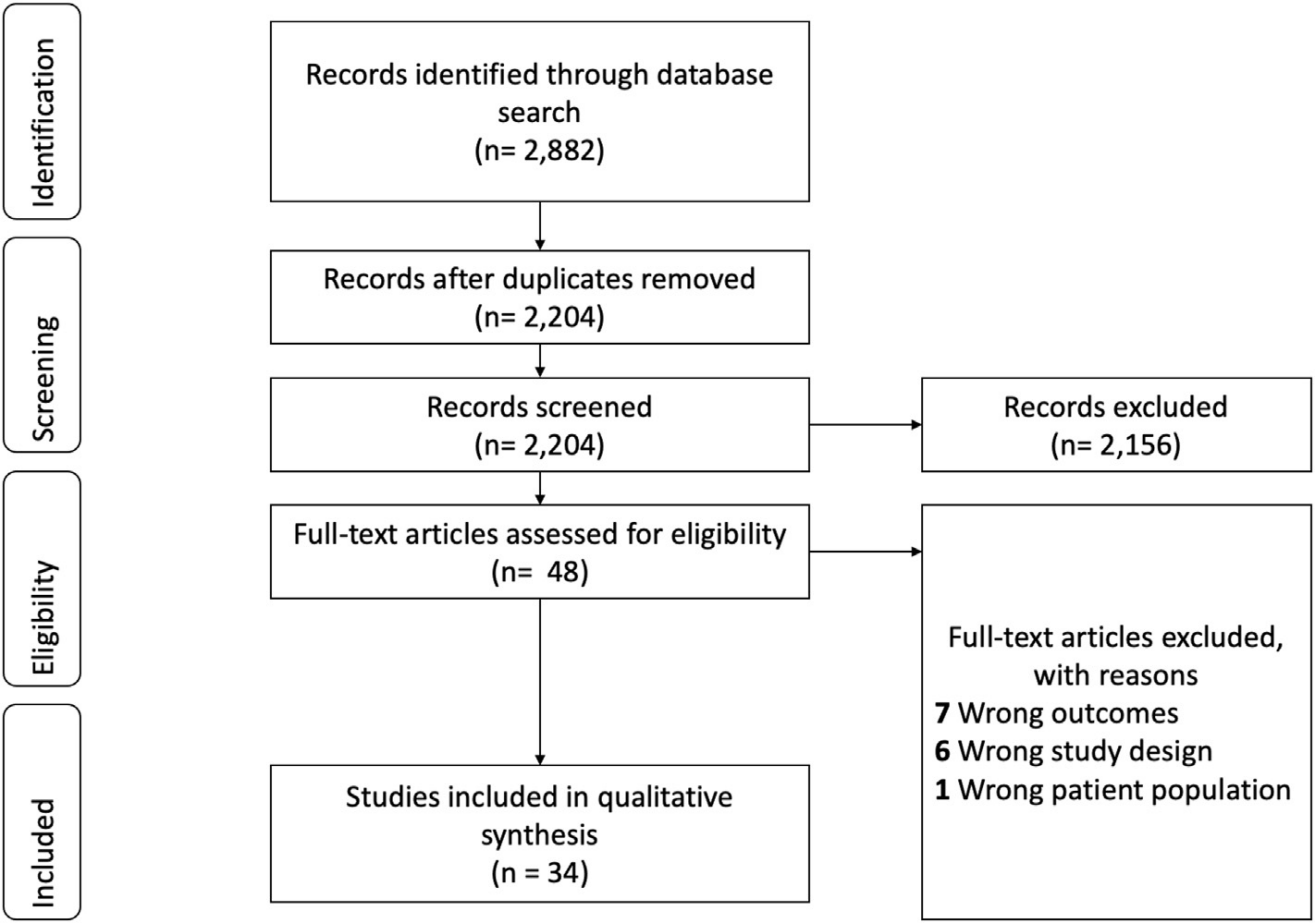
Systematic literature search flow diagram.

The initial search yielded 2882 studies. After removing 678 duplicates, 2204 studies were screened using the title and/or abstract. Full-text review was performed on 48 studies with 34 articles ultimately meeting the inclusion criteria for this study (Hwang et al., 2005, 2007; Samartzis et al., 2005; Cauthen et al., 1998; Schmieder et al., 2006; Rohe et al., 2009; Cabraja et al., 2012; Chen et al., 2013; Farrokhi et al., 2017; Kim et al., 2018; Moreland et al., 2004; Yamagata et al., 2012; Niu et al., 2010; Lee et al., 2014; Feng et al., 2018; Klingler et al., 2014; Yue et al., 2005; Yang et al., 2019; Moo et al., 2020; Hisey et al., 2015; Gornet et al., 2017; Mummaneni et al., 2007; Coric et al., 2011; Campbell et al., 2009). Results of the literature search and study selection process can be found in Fig. 1.

### 3.2 Study characteristics

Included studies stratified by fusion criteria and listed by the cohort graft type are found in Table 3.

**Table 3:** –Studies included in systematic review stratified by fusion criteria and graft material.

Author, Year	Rank	Design	Interbody	Mean Age	Follow-up (mo)	Levels (# of patients)	Spinal Level	Rates of			
								Fusion	Re-operation	Sub-sidence	Pseudo-arthrosis
Domagoj Coric et al., 2011	1	RCT	Allograft	43.9±7.39	24	1 (133)	C3 – C4: 3 C4 – C5: 6 C5 – C6: 83 C6 – C7: 41	82%	6%	0%	2.30%
Praveen Mummaneni et al., 2007	1	RCT	Allograft	43.9	24	1 (265)	C3 – C4: 3.8% C4 – C5: 5.7% C5 – C6: 56.2% C6 – C7: 34.3%	97.5%	1.9%	NR	NR
Hisey et al., 2015	1	RCT	Allograft	44	48	1 (81)	NR	94.4%	9.90%	NR	6.20%
Moo et al., 2020	1	PC	Allograft	52	36	2 (53)	C3–C5: 3 C4–C6: 14 C5–C7:36	96.15%	0%	11%	10%
Moo et al., 2020	1	PC	PEEK	56	36	2 (35)		100%	0%	30%	52%
Yang et al., 2019	1	PC	Allograft	50	29.43	1 (43); 2 (15)	C2 – C3: 2 C3 – C4: 10 C4 – C5: 14 C5 – C6: 29 C6 – C7: 18	100%	NR	NR	NR
Yang et al., 2019	1	PC	PEEK	50	30	1 (37); 2 (12)	C2 – C3: 2 C3 – C4: 8 C4 – C5: 9 C5 – C6: 30 C6 – C7: 12	100%	NR	NR	NR
Yue et al., 2005	1	RC	Allograft	52	86	1 (28); 2 (26); 3 (14); 4 (3)	NR	1 (96.4); 2 (88.5); 3 (71.4); 4 (66.7)	NR	47.90%	12.70%
Klingler et al., 2014	1	RC	PEEK	53	12	1 (27); 2 (12)	C3 – C4: 2 C4 – C5: 8 C5 – C6: 22 C6 – C7: 19	62.20%	2.60%	35.56%	NR
Lee et al., 2014	1	RC	PEEK	55	21	1 (78)	C3 – C4: 4 C4 – C5: 16 C5 – C6: 47 C6 – C7: 11	79.5%	NR	33.30%	NR
Yamagata et al., 2012	1	PC	Titanium	60	32	1 (31); 2 (32)	C3 – C4/C4 – C5: 35 C4 – C5/C5 – C6: 28	88.9%	NR	23.40%	NR
Majid R. Farrokhi et al., 2017	1	RCT	PEEK	45.28	12	1 (32)	C2 – C3: 1 C3 – C4: 3 C4 – C5: 6 C5 – C6: 15 C6 – C7: 7	93.8%	NR	NR	NR
Cabraja et al., 2012	2	RC	PEEK	57	26	1 (42)	C3 – C4: 6 C4 – C5: 10 C5 – C6: 20 C6 – C7: 6	88.1%	NR	14.28%	11.90%
Hwang et al., 2007	2	PC	Titanium	54	24	1 (3); 2 (20); 3 (4)	C3 – C4: 1 C4 – C5: 7 C5 – C6: 23 C6 – C7: 13	96.3%	NR	NR	NR
Rohe et al., 2009	2	PC	Titanium	48	87	1 (44); 2 (10)	NR	84%	NR	45%	NR
Cabraja et al., 2012	2	RC	Titanium	51	30	1 (44)	C3 – C4: 1 C4 – C5: 7 C5 – C6: 23 C6 – C7: 13	93.2%	NR	20.45%	6.80%
Schmieder, 2006	2	PC	Titanium	48	24	1 (54)	C3 – C4: 2 C4 – C5: 8 C5 – C6: 26 C6 – C7: 26 C7-T1: 2	98%	NR	45%	NR
Samartzis et al., 2005	2	RC	Allograft	45	12	1 (35)	NA^	100%	NR	NR	NR
Hwang et al., 2005	2	PC	Titanium	52	24	1 (14); 2 (28); 3 (21)	NR	97%	NR	3.8%	NR
Wang et al., 2020	3	RC	Allograft	56	39	1 (59); 2 (31); >/ ​= ​3 (17)	NR	1(96.6); 2(91.9); >/ ​= ​3 (88.9)	1 (0%); 2 (0%); >/ ​= ​3 (0%)	NR	1 (3.4%); 2 (8.1%); >/ ​= ​3 (11.1%)
Wang et al., 2020	3	RC	PEEK	53	39	1 (37); 2 (21); >/ ​= ​3 (3)	NR	1 (94.6%); 2 (92.9%); >/ ​= ​3 (90%)	1 (0%); 2 (9.5%); >/ ​= ​3 (33.3%)	NR	1 (5.4%); 2 (7.1%); >/ ​= ​3 (10%)
Chou et al., 2008	3	RC	PEEK	54.2	12	1 (3); 2 (6); 3(0)	NR	100%	NR	0%	NR
Wu et al., 2012	3	RC	Titanium	47.2	60	F1 (46); 2 (11)	C3 – C4: 5 C4 – C5: 14 C5 – C6: 36 C6 – C7: 13	100%	NR	NR	NR
Chou et al., 2008	3	RC	Titanium	55	12	1 (14); 2 (10); 3 (3)	NR	46.51%	NR	25.90%	NR
Shiban et al., 2016	3	RC	PEEK	55	12	1(127); 2 (125); 3 (13)	NR	90.67%	NR	26.19%	1.5%
Thomé et al., 2004	4	PC	Titanium	52±12	12	1 (13); 2 (5)	C4 – C5: 2 C5 – C6: 6 C6 – C7: 5 C4 – C6: 3 C5 – C7: 2	83.33%	0%	33.3%	0%
Arts et al., 2020	4	PC	PEEK	49	12	1 (48)	C3 – C4: 1 C4 – C5: 6 C5 – C6: 30 C6 – C7: 10 C7-T1: 1	90%	NR	NR	NR
Arts et al., 2020	4	PC	Titanium	50.3	12	1 (49)	C3 – C4: 6 C4 – C5: 2 C5 – C6: 21 C6 – C7: 19 C7-T1: 1	91%	4%	NR	NR
Thomé et al., 2006	4	RCT	Titanium	49	12	1 (37); 2 (13)	C3 – C4: 2 C4 – C5: 4 C5 – C6: 21 C6 – C7: 10 C3 – C5: 1 C4 – C6: 3 C5 – C:7 9	1 (71%); 2 (80%)	0%	18%	0%
Cho et al., 2004	5	PC	PEEK	53	12	2 (34); 3 (26)	C3 – C4: 24 C4 – C5: 34 C5 – C6: 56 C6 – C7: 32	100%	NR	NR	0%
Junaid et al., 2018	6	RC	PEEK	36	12	1 (59); 2(6)	C3 – C4: 1 C4 – C5: 7 C5 – C6: 41 C6 – C7: 10 C7-T1: 2 C3 – C5: 1 C4 – C6: 1 C5 – C:7 4	100%	NR	0%	NR
Cho et al., 2003	6	RCT	PEEK	53	12	1 (22); 2 (10); 3 (8)	C2 – C3: 1 C3 – C4: 9 C4 – C5: 18 C5 – C6: 26 C6 – C7: 12	100%	NR	NR	NR
Junaid et al., 2018	6	RC	Titanium	45.9	12	1 (68); 2(16)	C3 – C4: 2 C4 – C5: 8 C5 – C6: 40 C6 – C7: 16 C3– C5: 4 C4 – C6: 4 C5 – C:7 48	1 (100%); 2 (100%)	NR	0%	NR

Abbreviations: ACDF, anterior cervical discectomy and fusion; DDD, degenerative disc disease; NR, none reported; PC, prospective cohort; PEEK, polyetheretherketone; RC, retrospective cohort; RCT, randomized controlled trial.

### 3.3 Fusion rates

*Structural Allograft.* Seven studies (679 patients) used tier 1 or 2 criteria to assess fusion and reported an average fusion rate of 94.67% (range 82%–100%) after an average follow up of 37.1 months. One study used fibular allograft, 3 used an unspecified cortical allograft, and 3 used corticocancellous allograft (Samartzis et al., 2005; Yang et al., 2019; Moo et al., 2020; Hisey et al., 2015; Mummaneni et al., 2007; Coric et al., 2011). Among the three studies that used corticocancellous allograft, fusion rates at 24 months were 82%–96.2%. The reported fusion rates for those studies using unspecified cortical grafts were between 97.5% and 100%. The reported fusion rates for Yue et al., which used fibular allograft, was 92.59% at 86.4 months (Yue et al., 2005).

*PEEK Interbody Devices.* 275 patients with PEEK interbody devices were included in our final analysis. The average fusion rate was 87.27% (range 62.2%–100%) over average follow up of 12–36 months.

*Titanium Cages.* 279 patients with titanium cages were included in our final analysis including 5 prospective studies and 1 retrospective study. The average fusion rate was 92.86% (range 84%–100%) over average follow up of 37.1 months range (24–87 months).

### 3.4 Subsidence rate

*Structural Allograft.* Three studies of structural allograft reported subsidence rates. Coric et al. reported no cases of subsidence with 24 months follow-up (Coric et al., 2011). Moo et al. found a subsidence rate of 11.6% (6/53 patients) with 36 months follow-up (Moo et al., 2020). Yue et al. reported a subsidence rate of 47.9% (34/71 patients) at 86 months follow up (Yue et al., 2005).

*PEEK Cages.* Five studies reported subsidence rates. Klingler et all reported the highest subsidence rate occurring in 35.6% of levels (16/45) over 12 months (Klingler et al., 2014). Moo et al. reported 30% (21/70 levels) over 36 months (Moo et al., 2020). Lee et al. found 33.3% (26/78) over 21 months (Lee et al., 2014). Two other PEEK studies reported subsidence rates, Feng et al. at 0 and Cabraja et al. at 14.3% (6/42 patients) with follow up of 24 months and 26 months, respectively (Cabraja et al., 2012; Feng et al., 2018).

*Titanium Cages.* Of the three materials analyzed, titanium had the highest average subsidence rate of 27.5%. Five of the six analyzed studies reported subsidence rates with Rohe et al. and Schmieder et al. reporting the highest subsidence rates at 45% (24/54 patients in both studies) at 87 months and 24 months, respectively (Schmieder et al., 2006; Rohe et al., 2009). Hwang et al. recorded the lowest subsidence rate of the included studies at 3.8% (3/78 patients) at 25 months (Hwang et al., 2005). Yamagata et al. and Cabraja et al., reported subsidence rates of 23.4% (11/47 patients) and 20.5% (9/44 patients) at 32 months and 30 months, respectively (Cabraja et al., 2012; Yamagata et al., 2012).

### 3.5 Reoperation rate

*Structural Allograft.* Reoperation rates were available for 5 patient cohorts (Hisey et al., 2015; Gornet et al., 2017; Mummaneni et al., 2007; Coric et al., 2011; Campbell et al., 2009). The incidence of reoperation ranged from 0% to 9.9% (7/71 patients). The average reoperation rate across all analyzed studies including structural allograft was 6.2%.

*PEEK Cages.* Two studies of PEEK reported reoperation rates of 0% (Moo et al., 2020) and 2.6% (Klingler et al., 2014).

*Titanium Cages.* No studies of titanium cages, meeting the top 2 fusion criteria included in this study, reported reoperation rates.

### 3.6 Pseudarthrosis rate

*Structural Allograft.* Four allograft studies included in this systemic review reported pseudarthrosis rates. The lowest rate was reported as 2.3% (Coric et al., 2011) (3/133 patients) at 24 months while the highest rate was 12.7% (Yue et al., 2005) (9/71 patients) at 86 months. Across all studies reporting pseudarthrosis rates, the average rate was 7.8%.

*PEEK Cages.* Of the six PEEK studies two reported pseudarthrosis rates. Moo et al. reported 52% at 36 months and Cabraja et al. reported 11.9% at 26 months.

*Titanium Cages.* Pseudarthrosis rate was reported in 1 included study and found to be 6.8% at 30 months (Cabraja et al., 2012).

### 3.7 Patient reported outcomes

*Structural Allograft.* In studies using structural allograft, 5 reported NDI values. The improvement from pre- to post-operative NDI ranged from 62.1% to 79.6%. The average improvement in NDI across all analyzed studies including structural allograft was 66.2%. Four reported VAS values. Neck VAS improved between 71.1% and 94.6% and arm VAS improved between 77.4% and 92.3%. The average improvement for the neck and arm VAS were 80.5% and 83.4%, respectively.

*PEEK Cages.* In studies using PEEK, 3 reported NDI values. The improvement from pre to post-operative NDI ranged from 57.1% to 79.7% (average 69.1%). Two reported VAS values. Neck VAS improvement ranged from 43.8% to 79.6%. Arm VAS improvement ranged from 59.5% to 92.6%. The average improvement for the neck and arm VAS were 61.7% and 76.1%, respectively.

*Titanium Cages* In studies using titanium, only Chen et al. reported NDI values (Chen et al., 2013). The improvement from pre- to post-operative NDI for this study was 40.3%. Two studies reported VAS values, however for these only neck values were reported. For these neck VAS the improvement ranged from 63.8% to 65.9%. The average improvement for the neck VAS was 64.8%.

Study data including design, interbody, patient characteristics, fusion rates, and radiographic outcomes, are presented in Table 3.

## 4 Discussion

In this study, we systematically reviewed the literature for studies reporting fusion rates after ACDF using allograft, PEEK, or titanium cages in patients with degenerative cervical spinal conditions. To remove potential confounders, we focused on patients with either anterior plating or cages with integrated instrumentation (no stand-alone cages without instrumentation), and we excluded patients treated with BMP, platelets, or other agents designed to stimulate bone growth. At present, comparison of fusion by interbody type is difficult as standard methods for radiographic fusion assessment is not used (Table 2) (Tuchman et al., 2017; Oliver et al., 2018; Zadegan et al., 2017). In this study, we attempted to address this heterogeneity by ranking these methods according to predetermined criteria (Table 1). Ten studies (29%) used tier 1 fusion criteria and 6 (18%) used tier 2 criteria. Studies reporting tier 3–6 criteria do not provide enough information about the assessment of fusion to facilitate a meaningful comparison. We found a similar rate of fusion among structural allograft, titanium, and PEEK interbody cages. Of the 20 studies with tier 1 or 2 fusion criteria, the overall fusion rates after more than 1 year of follow-up, ranged from 62.2% to 100% for all graft types. The average fusion rates and ranges for the structural allograft, titanium, and PEEK studies were 94.67% (82%–100%), 92.9% (84%–100%), and 87.3% (62.2–100%).

Patient-reported outcomes were not uniformly collected or reported in studies assessing fusion after ACDF. In studies that did collect PROs, VAS and NDI were collected most frequently. However, differences in collection and reporting methodology prohibited meaningful direct comparison. Similarly, our other secondary outcomes measures (rates of pseudarthrosis, revision, and subsidence), were not commonly reported (Table 3). The methodologies associated with determining these metrics were often not reported or performed in a manner prohibitive to meaningful cross study comparison.

PEEK and titanium integrate with the osseous vertebral endplates to varying degrees. Both PEEK and titanium cages have undergone significant alterations since their inception resulting in evolving mechanical and morphologic properties (Papavero et al., 2002; Sugawara et al., 2011; Torstrick et al., 2017, 2018; Walsh et al., 2015). High-quality evidence in the form clinical trials comparing these modifications does not exist. As a result, we did not stratify within the titanium and PEEK cage groups for cage specific modifications.

The choice of interbody cage material may have a significant impact on healthcare cost. At present, studies on the cost of the various implants and comparative studies are lacking. Virk et al. evaluated the costs per quality adjusted life year (QALY) for ACDF using PEEK, allograft, or autograft (Virk et al., 2015) and found the cost per QALY to be $3228 for PEEK vs $2492 for allograft. Of note, in patients with myelopathy or chronic neck pain, all cage/graft choices were found to be cost-effective methods of improving outcomes. Cost-effectiveness or comparative studies involving titanium cages are lacking.

In this study, we focused on the use of ACDF for degenerative cervical indications. We found no differences in reported clinical and radiological outcomes between the graft materials. Outside the scope of the review, there may be other surgical goals (aside from fusion) warranting the use of a particular graft. For instance, in patients requiring close monitoring via follow-up MRI imaging, surgeons may opt for a graft choice which decreases MRI artifact (Stradiotti et al., 2009; Long et al., 2019). Additionally, for infectious surgical indications such as discitis/osteomyelitis, surgeon preference for the various cage types differ (Stradiotti et al., 2009; Burkhardt et al., 2019; Mondorf et al., 2009). For each sub-indication of ACDF, an optimal cage type may exist. Studies to address this important clinical question are needed.

### 4.1 Strengths and limitations

Several limitations are inherent in the study design. As with all systematic reviews, the present study is subject to publication bias. The number of studies, the heterogeneity of the study procedures, fusion criteria, and lack of studies directly comparing interbody materials, limited our attempts to perform a risk-of-bias analysis or meta-analysis.

The generalizability of our findings is limited by the inclusion and exclusion criteria that was applied. In many countries, ACDF is performed without anterior plating or with cages without integrated instrumentation. In this study, we did not include such studies to remove the confounding effects of anterior plating and/or integrated instrumentation. In doing so, we may have missed differences between graft materials which may be apparent when studied in the setting of entirely stand-alone grafts.

Lack of level 1 evidence comparing allograft vs PEEK vs titanium grafts limits the strength of the study conclusions. Additional high-quality evidence, in the form of prospective clinical trials with standardized fusion criteria, is needed. A significant strength of this systematic review was the reduction of heterogeneity in radiographic fusion assessment via the use of a prospectively determined fusion criteria ranking system. An additional strength is the comprehensive literature search and adherence to the PRISMA-guidelines.

This study focused on rate of fusion as the primary endpoint, however fusion rate is not the only goal of surgery. Depending on the indication, other important surgical goals include deformity correction and relief of neural element compression. As a result, it is possible that while fusion rate is similar across cage/graft types, certain cages may offer profound advantages in distinct situations. For example, synthetic cages may offer a superior result in the correction of severe deformity. Endpoints such as these could not be assessed in the present study but will be the subject of future studies.

## 5 Conclusion

Fusion rates after one or two level ACDF for degenerative cervical indications, are similar among titanium, PEEK, or structural allograft. High quality prospective randomized control trials comparing the interbody grafts is lacking in the peer reviewed literature. Additionally, the radiographic definition of fusion is highly variable. Studies using poorly validated or undescribed fusion metrics do not allow for comparison of fusion rates. After attempting to control for heterogeneity by stratifying studies by strength of fusion criteria and analyzing the best studies, we found no significant difference in fusion rate. Future high-quality studies using standardized and reliable fusion criteria are needed before clinical decisions based on fusion comparisons can be made. Future studies to assess surgical efficacy with regard to non-fusion endpoints (i.e. deformity correction) are needed and under way.

## Declaration of competing interest

The authors declare no relevant conflicts of interest or financial relationships affecting the impartiality of this work. In the interest of full disclosure of all financial relationships, the following authors disclose financial relationships: Dr. Roger Hartl - Consulting: DePuy Synthes, Brainlab, Ulrich, Royalties: Zimmer Biomet, Other: RealSpine (Investor). Dr. Dan Riew discloses royalties from Biomet, consulting fees from Nuvasive and Happe Spine, and stock interests in the following companies: axiomed, expanding orthopedics, spineology, spinal kinetics, amedica, vertiflex, benvenue, and paradigm spine. All other authors: none.
