# Supplementary material for: Titanium versus polyetheretherketone versus structural allograft in anterior cervical discectomy and fusion: A systematic review
Source: Brain Spine. 2022 Aug 22;2:100923. doi: 10.1016/j.bas.2022.100923 (PMC9560672; doi:10.1016/j.bas.2022.100923)
Supplement: Multimedia component 1 [file mmc1.doc]

**Appendix 1**

*Database searches and number of results*

Ovid MEDLINE (ALL: 1946 to present), Ovid EMBASE (1974 to present), and the Cochrane Library (Wiley) were searched from their inception through July 2021. There were no publication date, or article type restrictions on the search strategy. Non-English language articles and those published before 1990 were removed at the title and abstract screening. **Ovid MEDLINE(R**) In-Process & Other Non-Indexed Citations and **Ovid MEDLINE(R)** 1946 to Present were searched on 7/24/2021 yielding 662 references. **Embase (Ovid)** 1974 to Present was searched on 7/24/2021 yielding 1514 references. The Cochrane library was searched on 8/7/2021 with 123 references. Scopus was queried on 8/7/2021 with 523 references. In total this yielded 2882 references. After de-duplication, a total of 2204 references remained and were screened.

*Database search strategies*

**Ovid MEDLINE(R) In-Process & Other Non-Indexed Citations and Ovid MEDLINE(R) 1946 to Present – July 24, 2021**

1. exp Spinal Fusion/

2. (spin* adj2 fus*).ti,ab.

3. ("Anterior cervical discectomy" or spondylodes* or spondylosyndes*).ti,ab.

4. 1 or 2 or 3

5. exp Allografts/

6. (allograft* or homograft*).ti,ab.

7. ((allogeneic or homologous) adj2 (transplant* or graft*)).ti,ab.

8. exp Titanium/

9. titanium.ti,ab.

10. (Polyetheretherketone or PEEK).ti,ab.

11. 5 or 6 or 7 or 8 or 9 or 10

12. 4 and 11

**THE COCHRANE LIBRARY WILEY – AUGUST 07, 2021**

#1 MeSH descriptor: [Spinal Fusion] explode all trees

#2 (spin* near/2 fus*)

#3 ("Anterior cervical discectomy" or spondylodes* or spondylosyndes*)

#4 #1 or #2 or #3

#5 MeSH descriptor: [Allografts] explode all trees

#6 (allograft* or homograft*)

#7 ((allogeneic or homologous) near/2 (transplant* or graft*))

#8 MeSH descriptor: [Titanium] explode all trees

#9 titanium

#10 (Polyetheretherketone or PEEK)

#11 #5 or #6 or #7 or #8 or #9 or #10

#12 #4 and #11

**EMBASE (OVID) - JULY 24, 2021**

1. exp spine fusion/

2. (spin* adj2 fus*).ti,ab.

3. ("Anterior cervical discectomy" or spondylodes* or spondylosyndes*).mp.

4. 1 or 2 or 3

5. exp allograft/

6. (allograft* or homograft*).ti,ab.

7. ((allogeneic or homologous) adj2 (transplant* or graft*)).ti,ab.

8. exp titanium/

9. titanium.ti,ab.

10. (Polyetheretherketone or PEEK).ti,ab.

11. 5 or 6 or 7 or 8 or 9 or 10

12. 4 and 11

**SCOPUS - JULY 24, 2021**

TITLE-ABS-KEY ( ( ( spin* AND fus* ) OR "Anterior cervical discectomy" ) AND ( allograft* OR homograft* OR titanium OR polyetheretherketone OR peek ) )") OR (MH "Health Status Disparities")
